# Identifying microbial biomarkers of neurodegeneration: a comparative study in Alzheimer’s and Parkinson’s disease

**DOI:** 10.3389/frmbi.2026.1831956

**Published:** 2026-05-12

**Authors:** Simon De Jaegher, David Pinzauti, Maria D’Aguanno, Erika Parkinson, James Schofield, Fabio Strazzeri, Paul Skipp, Rebekah Penrice-Randal, Amy Kunicki, Beth McCausland, Christopher Kipps, Jay Amin, Manuele Biazzo

**Affiliations:** 1 The BioArte Ltd., Life Science Park, Triq San Giljan, San Gwann, Malta; 2 Laboratory of Molecular Microbiology and Biotechnology (LAMMB), Department of Medical Biotechnologies, University of Siena, Siena, Italy; 3 TopMD Precision Medicine Ltd, Southampton, United Kingdom; 4 Centre for Proteomic Research, University of Southampton, Southampton, United Kingdom; 5 Clinical Neurosciences, Clinical and Experimental Sciences, Faculty of Medicine, University of Southampton, Southampton, United Kingdom; 6 Memory Assessment and Research Centre, Hampshire and Isle of Wight Healthcare National Health Service (NHS) Foundation Trust, Southampton, United Kingdom; 7 Wessex Neurological Centre, Dept. Neurology, University Hospital Southampton, Southampton, United Kingdom

**Keywords:** Alzheimer’s disease, full-length 16S rRNA gene sequencing, gut microbiota, Parkinson’s disease, species-level resolution

## Abstract

**Introduction:**

Neurodegenerative disorders such as Alzheimer’s disease (AD) and Parkinson’s disease (PD) have been increasingly linked to alterations of the gut microbiota, although reported microbial signatures remain heterogeneous and often lack taxonomic resolution.

**Methods:**

In the present study, we applied full-length 16S rRNA gene sequencing to characterize gut microbiota composition in 152 individuals, including patients with AD (n = 37), PD (n = 65), and age-matched healthy controls (n = 50), using a unified bioinformatic and statistical framework with adjustment for relevant demographic covariates.

**Results:**

Alzheimer’s disease was associated with a modest but significant reduction in microbial richness and Shannon diversity compared with controls, whereas no alpha diversity differences were observed in PD. Beta diversity analyses revealed significant compositional differences across diagnostic groups, driven primarily by PD and modulated by sex but not age. Species-level differential abundance analysis identified a PD-associated microbial signature characterized by reduced abundances of short-chain fatty acid-producing bacteria, including *Faecalibacterium prausnitzii*, *Agathobacter rectalis*, *Roseburia intestinalis*, and *Faecalicatena fissicatena*, together with increased abundance of *Ruminococcus* sp. JE7A12. In contrast, AD exhibited minimal species-level changes, with only *Bacteroidales bacterium* CF showing reduced abundance compared with controls.

**Discussion:**

Overall, these findings indicate that Parkinson’s disease is characterized by a targeted disruption of beneficial butyrate-producing bacteria, whereas Alzheimer’s disease exhibits subtler and less consistent microbiome alterations. Our results underscore the importance of species-level resolution for identifying disease-associated microbial signatures.

## 1 Introduction

The human gut microbiota constitutes a complex and metabolically active ecosystem of bacteria, archaea, fungi, viruses, and protozoa that plays a critical role in host physiology (Matijašić et al., 2020). Beyond its well-established roles in digestion, nutrient processing (El Kaoutari et al., 2014), and immune homeostasis (Rooks and Garrett, 2016), emerging evidence indicate that gut microbial communities also influence central nervous system (CNS) function through the gut–brain axis (Zhu et al., 2017). This bidirectional network integrates immune signalling, microbial metabolites such as short-chain fatty acids (SCFAs), vagal transmission, and regulation of epithelial and blood–brain barrier integrity (Wikoff et al., 2009; Carabotti et al., 2015). Disruptions of this ecosystem, commonly referred to as dysbiosis (Winter and Bäumler, 2023), have been linked to several chronic conditions including metabolic disorders, inflammatory bowel disease, and neurodegenerative diseases (Dabke et al., 2019; Sun et al., 2021; Shan et al., 2022).

Neurodegenerative disorders such as Alzheimer’s disease (AD) and Parkinson’s disease (PD) represent a major global health burden, with rising prevalence and substantial clinical and socioeconomic burden (Ji et al., 2024). Both conditions are characterized by progressive neuronal dysfunction, long prodromal phases, and limited availability of early diagnostic biomarkers (Pfaffinger et al., 2025; Sharma et al., 2025). AD, the most prevalent form of neurodegeneration, is marked by cerebral accumulation of amyloid-β peptides and tau aggregates, driving inflammation, mitochondrial dysfunction, and ultimately neuronal loss (Liu et al., 2024). The incidence of AD rises sharply with age, affecting approximately 10% of individuals aged 65–75 and more than 30% of those over 80, with a continuing upward trajectory in prevalence (Seo et al., 2019; Askarova et al., 2020). The gut microbiota has long been suspected to contribute to AD pathogenesis. Evidence from mouse models suggests that age-, genetic-, and lifestyle-related shifts in microbiota composition promote the expansion of pro-inflammatory taxa (Zhang et al., 2021). This inflammatory state may alter cytokine production, increase intestinal permeability, and facilitate the leakage of microbial metabolites such as lipopolysaccharides and bacterial amyloids. These factors can influence blood-brain barrier integrity and modulate microglial and astrocyte responses, thereby affecting amyloid and tau pathology (Askarova et al., 2020; Chandra et al., 2023).

Lewy body diseases are the second most common group of neurodegenerative disorders after AD and are defined by pathological α-synuclein fibrils (Sekiya et al., 2025). They are progressively debilitating conditions characterized by α-synuclein fibrils (Lewy bodies) aggregating in the brain. The most common type of Lewy body disease is PD, which is typically diagnosed when motor symptoms such as bradykinesia, rigidity, and resting tremor become apparent. However, prodromal non-motor symptoms (including constipation, anosmia, and REM sleep behavior disorder) often precede the onset of motor features, reflecting early Lewy body pathology in the gut, olfactory system, and brainstem. At later stages, some PD patients develop dementia which can range from mild cognitive impairment to full-scale dementia (Nishiwaki et al., 2022; Ryman et al., 2023). The aetiology of PD remains incompletely understood, but are likely multifactorial, involving genetic predisposition, environmental influences, and perturbations in the gut microbiota (Omotosho et al., 2023). One prominent hypothesis proposes that α-synuclein aggregation may originate in the gastrointestinal tract, triggered by microbial or inflammatory stimuli, and subsequently propagate to the CNS via the vagal nerve (Braak et al., 2003). Although this theory remains under investigation, PD is strongly associated with constipation, intestinal dysbiosis, compromised mucosal barrier integrity (“leaky gut”), chronic inflammation, and small intestinal bacterial overgrowth (Wallen et al., 2022; Ryman et al., 2023).

Over the past decade, numerous studies provide converging evidence that gut dysbiosis is associated with both AD and PD (Pfaffinger et al., 2025). Case-control studies in PD consistently exhibit reduced abundances of SCFA-producing taxa such as *Faecalibacterium* and *Roseburia*, alongside increased levels of *Akkermansia*, *Ruminococcus*, and *Lactobacillus*, suggesting a relatively reproducible signature across cohorts (Li et al., 2023; Duan et al., 2024). SCFAs, particularly butyrate, propionate, and acetate, play critical roles in gut-brain communication: they maintain epithelial barrier integrity by nourishing colonocytes and modulating tight junctions via GPR41/43 signalling and HDAC inhibition (Fock and Parnova, 2023), and they regulate systemic and CNS inflammation, including microglial maturation (Erny et al., 2015) and anti-inflammatory polarization (Cao et al., 2025). A loss of these protective SCFA-producing microbes may therefore amplify neuroinflammatory pathways relevant to α-synuclein aggregation in PD and amyloid or tau pathology in AD (Harach et al., 2017; Chen and Lin, 2022). Conversely, enrichment of mucin-degrading or pro-inflammatory taxa may predispose to gut barrier dysfunction, systemic endotoxemia, and downstream neuroimmune activation (Forsyth et al., 2011). In contrast, findings in AD are more heterogeneous, with smaller effect sizes and limited overlap across studies (Dissanayaka et al., 2024). Variability in sample collection, sequencing depth, geographic factors, medication exposure, and taxonomic resolution likely contribute to inconsistencies and complicate efforts to identify robust biomarkers (Lampeter et al., 2023).

Most studies rely on 16S rRNA sequencing or analyses aggregated at the genus or family level, limiting insights into species- and strain-level differences with functional or diagnostic relevance. Direct comparisons between AD and PD within the same analytical framework are rare, making it difficult to determine whether observed signatures are disease-associated or reflect shared aging- or medication-related patterns. Additionally, potential modulators such as demographic variables remain insufficiently explored despite emerging evidence of their influence on microbial composition (Heravi et al., 2023).

Given these limitations, high-resolution microbial profiling is increasingly recognized as essential for identifying disease-associated signatures and informing the development of targeted microbiome-modulating strategies. Because AD and PD are heterogeneous conditions with diverse underlying biological processes, species-level resolution is particularly important. Detailed taxonomy and functional profiling may help distinguish mechanistically relevant subtypes and support precision-medicine approaches (Kern et al., 2025).

In this study, we address these gaps by applying high-resolution full-length 16S rRNA gene sequencing to characterize gut microbial communities at species-level resolution in patients diagnosed with AD and PD, together with rigorously age-matched healthy controls. Our objectives were to (i) characterize gut microbiota diversity and species-level community composition across neurodegenerative disorders and healthy controls; (ii) identify robust microbial signatures associated to AD and PD, as well as potential overlaps; and (iii) evaluate the influence of demographic and clinical variables microbial abundance patterns. By integrating unified sequencing and analytical workflows across all groups, this work aims to refine current understanding of gut microbiota alterations in neurodegeneration and support the identification of reproducible microbial biomarkers and biologically relevant mechanisms.

## 2 Materials and methods

### 2.1 Participants and data collection

Participants were recruited between February and September 2022 by two clinical research sites: the Memory Assessment and Research Centre (MARC) at Moorgreen Hospital Southern Health NHS Foundation Trust, UK and the Neurodegenerative Research Team at University Hospitals Southampton NHS Foundation Trust, UK. Recruitment involved screening individuals from local research databases, the Join Dementia Research (JDR) online platform, and clinician referrals.

Eligible participants were adults aged between 50 and 85 years who were able and willing to provide informed consent. Individuals in the disease groups met established consensus diagnostic criteria: participants with AD fulfilled the National Institute of Neurological and Communicative Disorders and Stroke and the Alzheimer’s Disease and Related Disorders Association (NINCDS-ADRDA) criteria (Dubois et al., 2007) for probable AD, while participants with PD met the Movement Disorders Society (MDS) criteria (Postuma et al., 2015) for PD. Exclusion criteria included lack of capacity or unwillingness to consent, concurrent participation in a clinical trial involving investigational drugs, diagnosis of mixed dementia or other central nervous system diseases (e.g., multiple sclerosis, stroke), and insufficient English proficiency.

Control participants were required to have no cognitive impairment and a Montreal Cognitive Assessment (MoCA) score greater than 26 (Davis et al., 2021). To ensure comparability across diagnostic groups, control participants were matched to AD and PD cases based on age. This design enabled robust between-group comparisons while minimizing confounding factors related to demographic variability.

The study was conducted under the framework of the RAS project (ID: 303109) and sponsored by the University of Southampton, UK. Ethical approval of the study was obtained from the West Midlands – South Birmingham Research Ethics Committee (reference: 21/WM/0252). Institutional approval was also granted by the University of Southampton Ethics and Research Governance Online (ERGO) system (reference: 66828). All participants provided written informed consent prior to enrolment, and the study adhered to the principles of the Declaration of Helsinki, Good Clinical Practice (GCP), the Data Protection Act (2018), and the Mental Health Capacity Act (2005).

Following consent procedures, participants attended a single study visit to collect demographic and clinical information. The Hoehn and Yahr scale was utilized to quantify motor symptoms associated with PD, and cognition was assessed using the MoCA. Stool samples were collected at participant’s homes with DNA/RNA Shield-Fecal Collection Tube (Zymo Research, Irvine, CA, USA). The samples were returned to the recruitment centers and frozen before shipping on dry ice to The BioArte Ltd laboratories (San Gwann, Malta) where they were stored at -80 °C prior to processing.

### 2.2 Full-length 16s rRNA sequencing

Total DNA was extracted using the MagMax™ Microbiome Ultra Nucleic Acid Isolation Kit (Applied Biosystems, Carlsbad, CA, USA) in combination with the KingFisher™ Flex Purification System (Thermo Fisher Scientific, Waltham, MA, USA) following manufacturer’s guidelines. Mechanical lysis was performed via bead-beating at 6 m/s for 40 seconds using the MP FastPrep-24™ 5G homogenizer (MP Biomedicals, Irvine, CA, USA). Quantification of extracted DNA was performed with the Qubit™ 4 Fluorometer (Thermo Fisher Scientific) using the 1X dsDNA High Sensitivity Assay Kit.

Full-length 16S rRNA genes (~1500 bp) were amplified using primers 27f (5′ -TTTCTGTTGGTGCTGATATTGC-AGRGTTYGATYMTGGCTCAG-3′) and 1492r (5′ -ACTTGCCTGTCGCTCTATCTTC-CGGTTACCTTGTTACGACTT-3′). PCR reactions were set up in 12.5 µL volumes containing 6.25 µL LongAmp^®^ Taq 2X Master Mix (New England Biolabs, Ipswich, MA, USA), 400 nM of each primer, and 4.25 µL of template DNA. Amplification was carried out on a T100™ thermal cycler (Bio-Rad, Hercules, CA, USA) under the following cycling conditions: initial denaturation at 95 °C for 4 min; followed by 25 cycles at 95 °C for 20 s, 51 °C for 30 s and 65 °C for 4 min; and a final extension 65 °C for 5 min. PCR yields were evaluated by electrophoresis using E-Gel™ Double Comb 2% Agarose Gels with SYBR™ Safe DNA Gel Stain (Invitrogen, A42348). Amplicons were purified using Agencourt^®^ AMPure^®^ XP beads (Beckman Coulter, Indianapolis, IN, USA). A 0.6× bead-to-sample ratio was used, with 5 min incubation at room temperature. Beads were immobilized on a magnetic rack (NimaGen, Nijmegen, Netherlands), washed twice with 70% ethanol, and eluted in 15 µL of nuclease-free water. After a second 10 min incubation, eluates were collected and quantified using a NanoDrop™ 8000 Spectrophotometer (Thermo Fisher Scientific).

Sample-specific barcodes were introduced through a second PCR using a modified version of the PCR Barcoding Expansion 1–96 kit (Oxford Nanopore Technologies, ONT). A custom set of 5′-phosphorylated primers was used to eliminate the need for end-prep and dA-tailing, reducing time and cost. The reaction mix (25 µL) included 12.5 µL LongAmp Taq 2X Master Mix, 0.5 µL of 10 µM primer mix, and 100 fmol of the purified 16S amplicon. PCR cycling was performed on a T100 thermal cycler under the following conditions: initial denaturation at 95 °C for 3 min; 12 cycles of 95 °C for 15 s, 62 °C for 15 s, and 65 °C for 4 min; and a final extension at 65 °C for 15 min. Barcoded products were verified on a 2% agarose gel, purified using 0.6× AMPure XP beads (as above), and quantified via NanoDrop 8000. Amplicons were pooled to a final volume of 30 µL containing 500 ng of DNA.

Sequencing adapters were ligated using the Ligation Sequencing Kit SQK-LSK114 (ONT). To the 30 µL DNA pool, 12.5 µL Ligation Buffer (LNB), 5 µL Quick T4 DNA Ligase, and 2.5 µL Ligation Adapters (LA) were added. The mix was incubated for 10 min at room temperature. Following ligation, 0.4× AMPure XP beads were added and incubated for 5 min. Beads were washed twice with 250 µL Short Fragment Buffer (SFB) and resuspended in 15 µL elution buffer. After a final 10 min incubation, eluates were collected in DNA LoBind tubes (Eppendorf). Library loading and flow cell priming were performed per ONT protocols. A total of 10 fmol of the prepared DNA library was loaded dropwise onto an R10.4.1 flow cell, and sequencing was carried out on a GridION Mk1B device (ONT), enabling real-time, super-accurate basecalling and live demultiplexing using default settings.

### 2.3 Data analysis workflow

Native pod5 files generated by Oxford Nanopore sequencing were live basecalled and demultiplexed by Guppy v.6.1.5 (Oxford Nanopore Technologies), using the super-accurate model and a minimum quality score of 12. Read quality and length distributions were inspected with NanoPlot v.1.44.1 (De Coster and Rademakers, 2023). The sequencing FASTQ reads were processed with a custom bioinformatic pipeline. The pipeline trimmed off sequencing adapters and primer sequences with cutadapt v.5.0 (Martin, 2011), then filtered out reads based quality and length (DADA2 R package v.1.22.0) (Callahan et al., 2016). Only reads between 1300 and 1600 nucleotides in length and with a maximum expected error rate of 25 (maxEE = 25) were retained. Potential chimeric reads were identified and removed with a *de novo* approach applying minimap2 v.2.28 (-x ava-ont preset and a maximum seed distance of 500) (Li, 2018) and yacrd v.1.0.0 run with default settings (Marijon et al., 2020). Taxonomy was inferred by emu v.3.5.2 (Curry et al., 2022) comparing each reads to a curated taxonomic reference database (Curry et al., 2022) to assign species-level classification. The resulting tab-separated output was subsequently imported into R for downstream statistical analyses.

### 2.4 Statistical analysis

All statistical analyses were carried out in R (v. 4.5.0) (R Core Team, 2010) within the RStudio (v. 421) (Posit team, 2023) environment. Group-level comparisons of demographic and clinical variables were conducted using Chi-square tests for categorical variables and Kruskal–Wallis tests for continuous variables, followed by *post-hoc* pairwise Wilcoxon rank-sum tests with Benjamini–Hochberg correction where appropriate. A significance threshold of *p* < 0.05 was applied throughout all analyses.

To minimize the influence of low-abundance taxa, the dataset was filtered to retain only those taxa with at least 10 counts in more than 0.7% of the samples (≥2 samples) using the filter_taxa function from *phyloseq* (v. 1.52.3) (Paul, 2017). For diversity analyses, the filtered dataset was rarefied to a uniform sequencing depth of 25,000 reads per sample using the rarefy_even_depth function from *phyloseq*. Alpha diversity was quantified using the Shannon and Simpson indexes, as well as the observed richness (number of unique taxa), computed with *phyloseq*. Differences between groups were assessed using Generalized Linear Models (GLMs) from the *stats* package (v. 4.5.0). Additional pairwise comparisons were carried out using the emmeans function from the *emmeans* package (v. 1.11.2) (Lenth, 2017). Beta diversity analyses were performed on Hellinger-transformed counts (using *microbiome* v. 1.30) (Lahti and Shetty, 2017) and visualized through ordination via Principal Coordinates Analysis (PCoA) based on Bray–Curtis dissimilarities. Plots were generated using *microbiome* and *ggplot2* (v. 3.5.2) (Wickham et al., 2007; Paul, 2017). Community-level differences were tested using PERMANOVA with the adonis2 function from *vegan* (v. 2.6.10) (Oksanen et al., 2001). Pairwise group comparisons were further conducted with the pairwise.adonis2 function from *pairwiseAdonis* (v. 0.4.1) (Martinez Arbizu, 2020), and group homogeneity of dispersion was examined using betadisper followed by permutest in *vegan* (Oksanen et al., 2001).

Differential abundance analysis at the species level was performed using ANCOM-BC2 (v2.10.1) (Lin and Peddada, 2024). Analyses were run on filtered bacterial species abundance tables, applying a prevalence cut-off of 10% and a library size threshold of 1000 reads. The fixed-effects model included Patient_group, Age, and Sex, while structural zeros were accounted for and log-fold change shrinkage was applied. P-values were adjusted using the Benjamini-Hochberg method, with a significance threshold of *q* < 0.05. Species with an absolute log_2_-fold change > 0.58 (≥ 1.5-fold change in abundance) in at least one comparison were considered significant, consistent with a prior microbiome study that applies this cutoff to focus on biologically meaningful abundance differences rather than marginal effect sizes (Rodriguez et al., 2024). For visualization and reporting purposes, only species reaching at least 1% relative abundance in at least 10% of samples were retained.

To investigate associations between disease progression (disease duration), cognitive function (MoCA score), motor severity (Hoehn and Yahr score), and gut microbiota composition, we performed Spearman correlation analyses between each clinical variable and the relative abundance of microbial species, separately within AD and PD cohorts. Species were filtered to retain only those with a minimum prevalence of 10% across samples and mean relative abundance ≥ 0.01%, ensuring robust statistical testing. Multiple testing correction was applied using the Benjamini-Hochberg false discovery rate (FDR) method, with a significance defined at FDR ≤ 0.2.

## 3 Results

### 3.1 Study population

**Figure 1:**
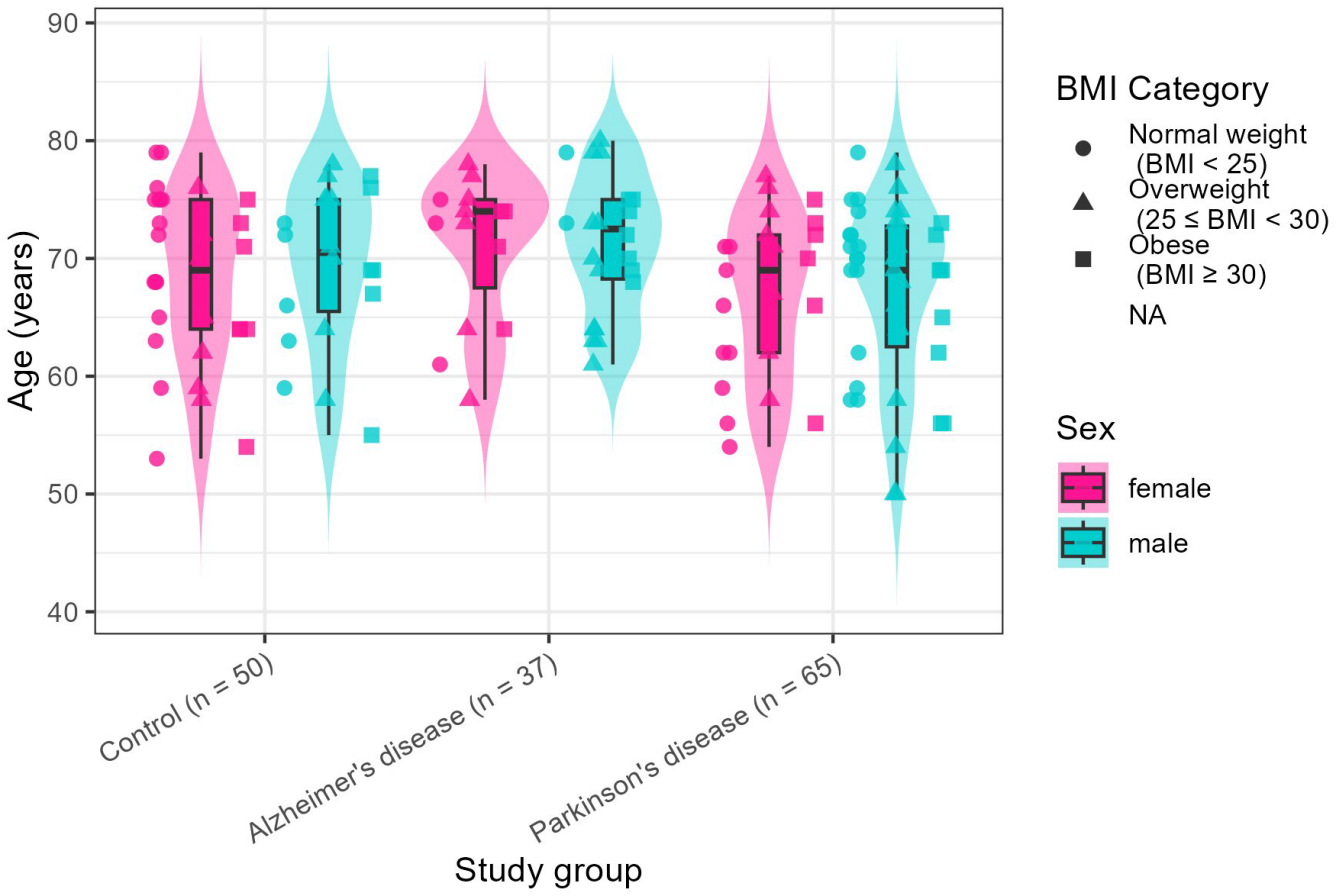
Demographic and clinical distribution of participants across study groups. Violin plots illustrate the age distribution within AD, PD, and healthy control groups. Data are stratified by sex, with female participants represented in pink and male participants in blue. Individual body mass index (BMI) categories are represented by distinct shapes.

A total of 152 individuals were recruited in this study, including 37 participants with Alzheimer’s disease (AD), 65 with Parkinson’s disease (PD), and 50 age-matched healthy controls (**Supplementary Table S1**). The median age of the cohort was 69 years. Participants with AD were significantly older than those with PD (*p* = 0.013), whereas no substantial age difference was observed between PD and control participants and between AD and control participants (**Table 1**). The AD and PD groups showed a higher proportion of males (59.5% and 64.6%, respectively), while the control group was predominantly female (60%). Notably, the male predominance observed in the AD cohort contrasts with established epidemiological trends, in which females are typically more affected, whereas the male predominance in PD is consistent with previous reports (Nicoletti et al., 2023). Similar body mass index (BMI) distributions were observed across all groups (**Figure 1**). Cognitive performance, assessed using the Montreal Cognitive Assessment (MoCA), differed significantly between groups. Healthy controls showed preserved cognition (mean ± SD: 28 ± 1.2), participants with Parkinson’s disease exhibited moderate impairment (25.1 ± 3.6), and those with Alzheimer’s disease showed severe impairment (16.3 ± 6). Disease duration was longer in PD participants (8.1 ± 6 years) compared with AD participants (2.6 ± 2.5 years). Medication use and constipation status are provided in **Supplementary Table S1**.

**Table 1:** Demographic and clinical characteristics of study participants.

Variable	Control (n = 50)	Alzheimer (n = 37)	Parkinson (n = 65)
Sex (M/F)	20/30	22/15	42/23
Age (mean ± SD)	68.8 ± 7	71.3 ± 5.9	67.2 ± 7.3
BMI (mean ± SD)	27.6 ± 5.4	28.1 ± 4.3	26.7 ± 5
Ethnicity (White/others)	50/0	36/0 (1 NA)	59/6
Handedness (R/L)	43/7	31/5 (1 NA)	59/6
Years of education	14.2 ± 3.7	14.6 ± 3.8	14 ± 3.4

Summary of age, sex, BMI, ethnicity, handedness (right or left), and education across control, AD, and PD patients. Data are shown as mean values ± standard deviation (SD).

To evaluate potential confounding effects, demographic variables were formally compared between groups. Significant differences were observed for age (*p* = 0.017) and sex (*p* = 0.026), whereas BMI (*p* = 0.405) and ethnicity (*p* = 0.213) did not differ significantly. Accordingly, age and sex were included as covariates in all subsequent alpha- and beta-diversity analyses. MoCA score and disease duration were not included as covariates, as they reflect core clinical features and disease progression indicators rather than independent confounding factors.

### 3.2 Sequence data quality and filtering

The raw full-length 16S rRNA amplicon sequencing dataset contained over 14.3 million reads, with an average Q-score 17.2 and an average read length of 1,667.5 bp (N50 = 1,642 bp). Reads that did not meet quality criteria, including chimeric sequences or those outside the 1,300–1,600 bp range, were removed. After filtering, the dataset retained approximately 8.57 million high-quality reads, with each of the 152 samples contributing an average of 56,361 reads (min: 25,722; max: 90,950). The remaining reads had an average length of 1,448.3 bp (N50 = 1,449 bp) and an average Q-score of 21.2.

The high-quality reads were subsequently classified using emu to assign taxonomy. Given this distribution, the minimum sequencing depth measured (25,722 reads) was used as a reference and the dataset was rarefied to 25,000 reads per sample to ensure consistent sequencing depth for downstream diversity analyses, retaining 44.3% of the high-quality sequencing reads. Sparsity decreased slightly after rarefaction (from 0.774 to 0.713), and approximately 78.1% of species were retained across the dataset, reflecting a minor loss of low-abundance taxa.

### 3.3 Richness, evenness, and diversity metrics

**Figure 2:**
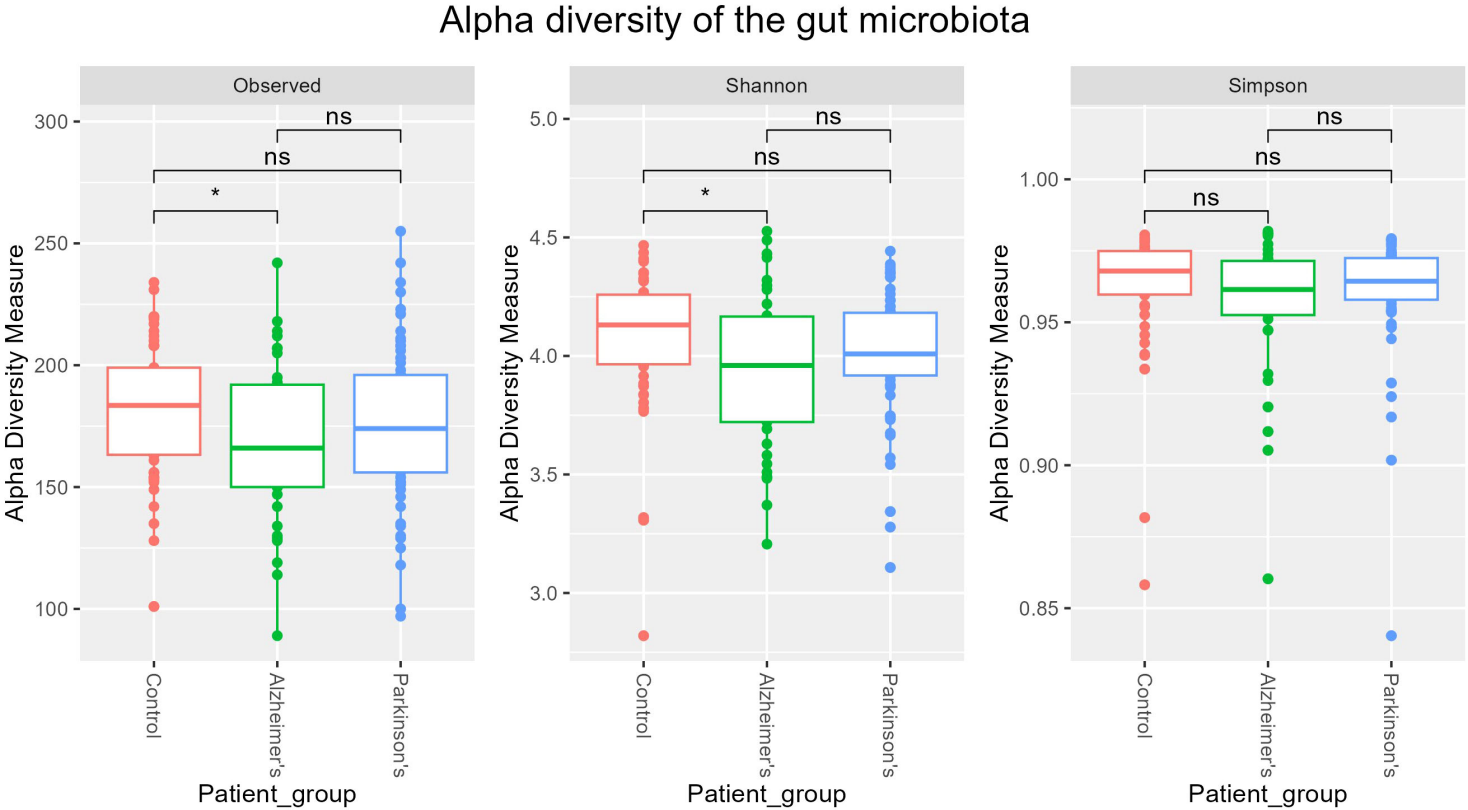
Species-level alpha diversity. Boxplots show Observed richness, Shannon index, and Simpson index for controls (red), AD (green), and PD (blue) patients. Statistical significance was assessed using GLM model including age and sex as covariates. Asterisks (*) indicate statistically significant p-values (*p* < 0.05), while ns indicates non-significant differences.

Statistical testing was performed on rarefied data using generalized linear models with age and sex included as covariates; neither variable was identified as a confounder (*p* > 0.05). Significant difference in alpha diversity was identified between Alzheimer’s patients and healthy controls (**Figure 2**). AD patients showed a modest yet significant decrease in microbial diversity (Shannon index, *p* = 0.027) and richness (observed species, *p* = 0.030), while no statistically significant differences were measured for PD patients. Simpson index showed no statistically significant differences among study groups.

**Figure 3:**
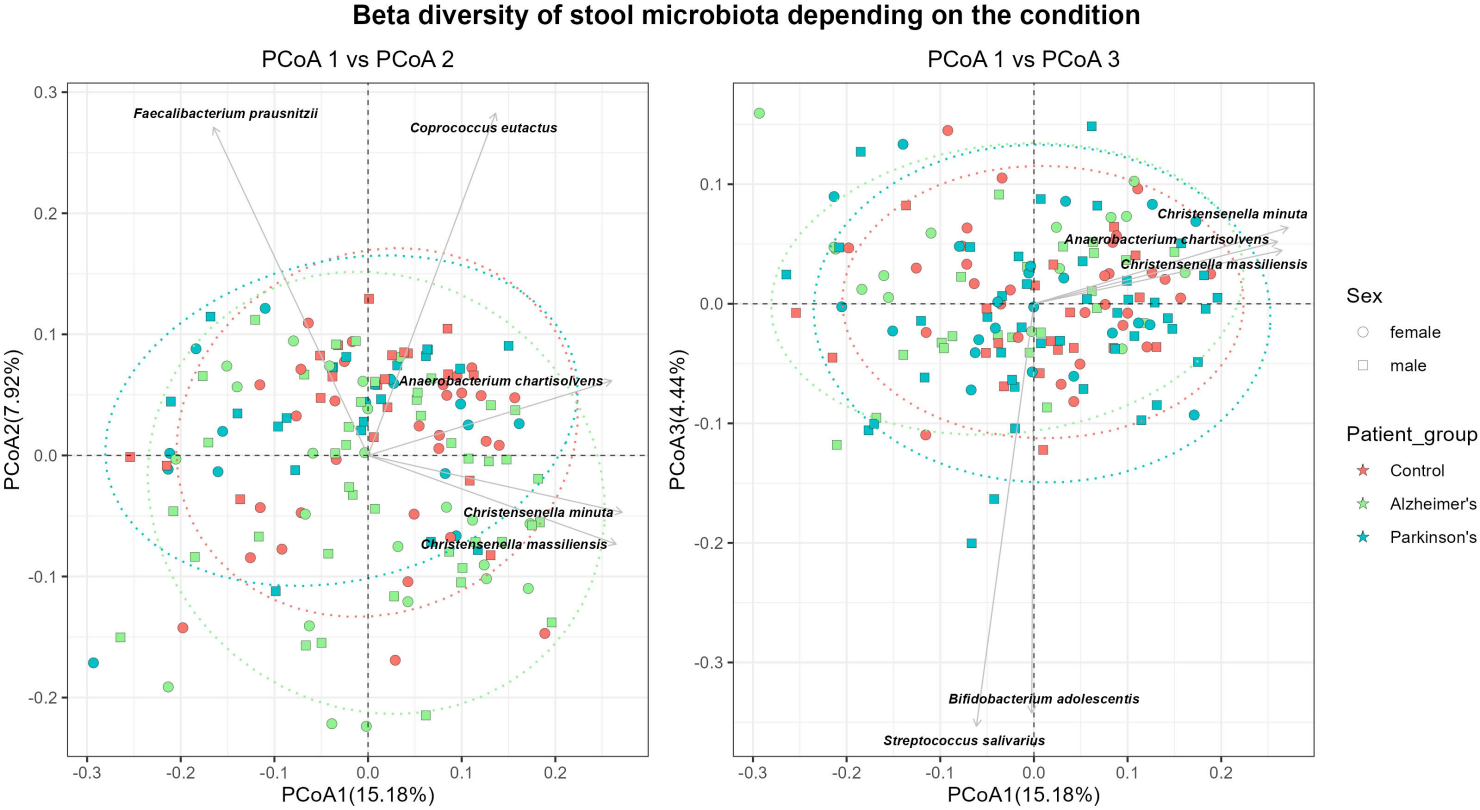
Beta diversity across study groups. Principal coordinate analysis (PCoA) plots showing beta diversity based on Bray-Curtis distance computed on rarefied, Hellinger-transformed data. Samples are colored based on study group (controls, red; AD, green; and PD, blue) and shaped by sex (circles, male; squares, female). Selected species are shown as arrows, indicating taxa that contribute mostly to variation along the principal coordinates.

Beta diversity was assessed using Bray–Curtis dissimilarities on rarefied and Hellinger-transformed data, visualized by PCoA (**Figure 3**). PERMANOVA analysis, including sex and age as covariates, revealed significant differences in microbial community composition between study groups (*p* = 0.001, R^2^ = 0.026), although there was a large degree of overlap in the 95% confidence ellipses. Sex also showed a significant association with beta diversity (*p* = 0.012, R^2^ = 0.012), while age had no detectable effect (*p* = 0.262). Pairwise comparisons indicated significant differences between Controls and Parkinson’s patients (*p* = 0.047) as well as between AD and PD patients (*p* = 0.005). Assessment of group dispersion indicated significant heterogeneity among patient groups (*p* = 0.024), implying that part of the PERMANOVA signal may be driven by differences in variance rather than compositional shifts alone. Pairwise testing showed no dispersion differences between Controls and AD patients (*p* = 0.55) and between AD and PD patients (*p* = 0.095), whereas significant heterogeneity was detected between Controls and PD patients (*p* = 0.009). In contrast, dispersion was not significant for sex (*p* = 0.54), implying that the association of sex with microbial structure reflects a biologically meaningful effect rather than an artifact of dispersion differences.

To assess the potential confounding effect of pharmacological treatments, multivariate PERMANOVA models were performed incorporating medication classes (**Supplementary Table S1**) as covariates. Medication groups strongly associated with disease status such as antiparkinsonian treatments, acetylcholinesterase inhibitors, and memantine, were excluded due to high collinearity with diagnostic categories, which would preclude reliable estimation of their independent effects. In contrast, medication classes more evenly distributed across groups, including statins and nonsteroidal anti-inflammatory drugs (NSAIDs), were retained in the model. In the adjusted analysis, disease status remained significantly associated with gut microbiota composition (PERMANOVA, R^2^ = 0.0219, *p* = 0.004), suggesting that the observed differences are not solely related to the included medication variables. Sex also showed a significant effect (R^2^ = 0.0113, *p* = 0.015), whereas age was not significantly associated with microbial composition (R^2^ = 0.1618, *p* = 0.818). Neither statin use (R^2^ = 0.0079, *p* = 0.188) nor NSAIDs use (R^2^ = 0.0057, *p* = 0.698) was significantly associated with microbiota variation.

### 3.4 Gut microbiome composition

**Figure 4:**
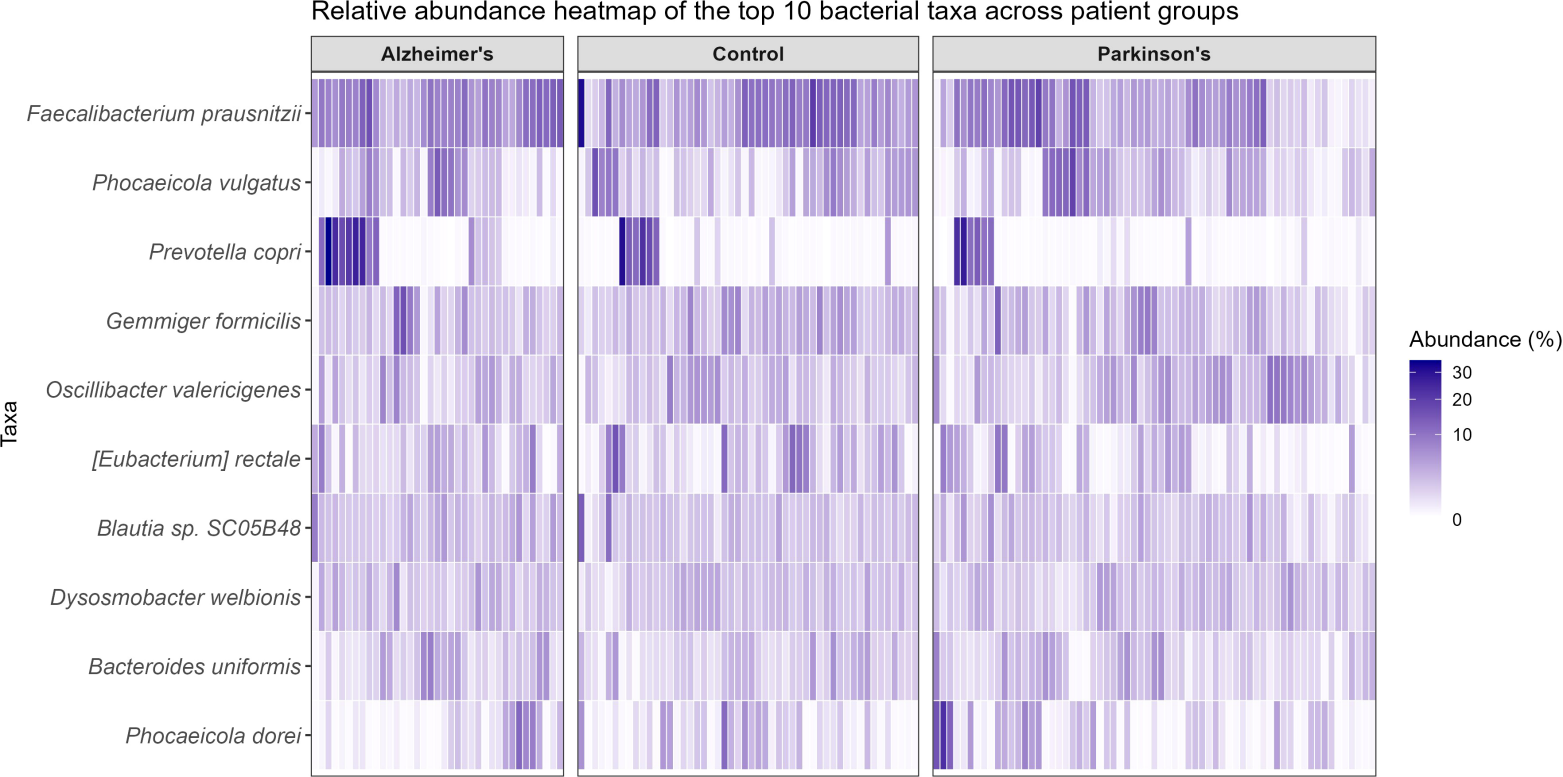
Relative abundance of the ten most abundant taxa across groups. Heatmap displaying the relative abundances of the ten most abundant bacterial taxa across patients. Taxa are reported on the Y-axis, while individual samples are represented on the X-axis, grouped by condition (control, AD, or PD). The color gradient represents measured relative abundance (%), with darker shades indicating higher abundances.

The sequencing reads were classified into 785 bacterial species representing 321 genera. Taxonomically unassigned reads represented 0.53% of the total dataset (45,684 reads overall; ranging from 300 to 441 reads per sample) and were excluded from downstream analyses. Microbial communities in the Control, AD, and PD groups were dominated by the phyla *Firmicutes* (73.2%, 72.4%, and 73.7%, respectively) and *Bacteroidota* (19.6%, 21.9%, and 18.8%, respectively), together representing over 90% of the classified sequencing reads. A heatmap illustrating the relative abundance of the 20 most abundant species across the dataset is shown in **Figure 4**, highlighting the differences between study groups. Across all samples, the ten most abundant bacterial species were *Faecalibacterium prausnitzii* (7.09% of the total community), *Phocaeicola vulgatus* (3.07%), *Prevotella copri* (2.83%), *Gemmiger formicilis* (2.82%), *Oscillibacter valericigenes* (2.69%), *Agathobacter rectalis* (2.26%), *Blautia* sp. SC05B48 (2.21%), *Dysosmobacter welbionis* (2.18%), *Bacteroides uniformis* (1.91%), and *Phocaeicola dorei* (1.72%). Mean relative abundances stratified by clinical group are summarized in **Table 2**.

**Table 2:** Mean relative abundance of the ten most abundant bacterial species across study groups.

Taxa	Control	Alzheimer’s	Parkinson’s
*Faecalibacterium prausnitzii*	7.91 ± 5.45	8.04 ± 3.60	5.92 ± 4.81
*Phocaeicola vulgatus*	3.19 ± 3.28	2.98 ± 3.58	3.03 ± 3.66
*Prevotella copri*	2.44 ± 6.42	5.38 ± 9.54	1.68 ± 5.19
*Gemmiger formicilis*	3.07 ± 1.91	2.92 ± 3.32	2.58 ± 2.39
*Oscillibacter valericigenes*	2.40 ± 1.81	2.27 ± 1.97	3.15 ± 2.60
*Agathobacter rectalis*	2.88 ± 3.65	2.27 ± 2.18	1.77 ± 2.42
*Blautia* sp. SC05B48	2.35 ± 2.26	2.61 ± 1.64	1.89 ± 1.32
*Dysosmobacter welbionis*	2.15 ± 1.05	2.27 ± 1.47	2.16 ± 1.22
*Bacteroides uniformis*	1.78 ± 1.50	2.17 ± 2.17	1.87 ± 1.82
*Phocaeicola dorei*	1.48 ± 2.23	1.47 ± 2.71	2.05 ± 3.87

### 3.5 Differential abundance analysis

**Figure 5:**
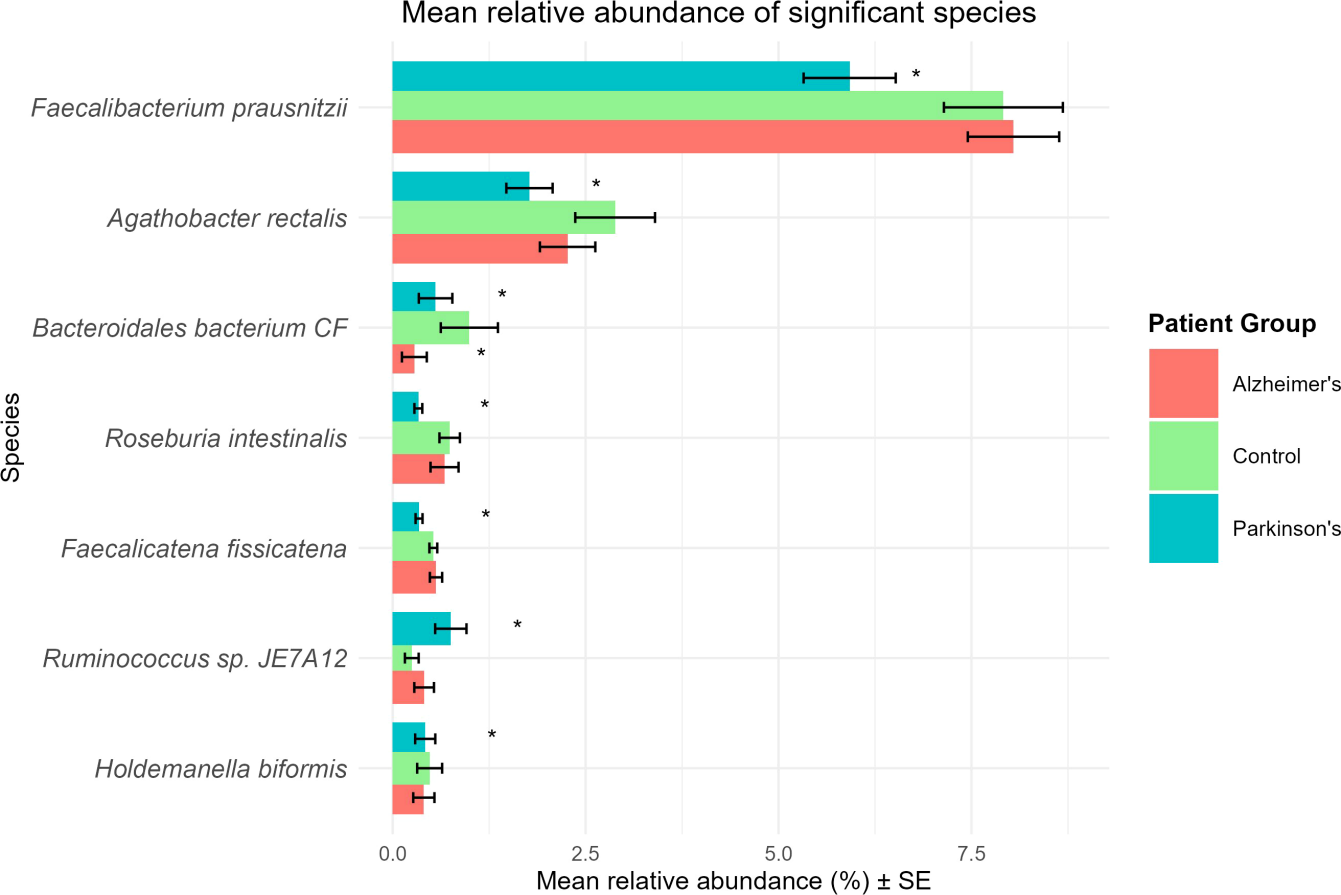
Relative abundance of differential abundant species across patient groups. Boxplot showing the mean relative abundance ± standard error (SE) of target DA across healthy controls (red), AD (green), and PD (blue) patients. Asterisks (*) indicate groups with statistically significant difference compared with controls.

Differential abundance testing was performed using ANCOM-BC2. A total of seven species exhibited significant changes in at least one patient group (*q* < 0.05; |log_2_FC| > 0.58) (**Table 3**). The bacterial community of the stool samples of PD patients was distinguished from that of healthy controls by the higher abundance of *Ruminococcus* sp. JE7A12 (increased log_2_FC) and lower abundances of *Faecalibacterium prausnitzii*, *Agathobacter rectalis*, *Roseburia intestinalis*, *Faecalicatena fissicatena*, *Holdemanella biformis*, and *Bacteroidales bacterium* CF (decreased log_2_FC). Interestingly, *Bacteroidales bacterium* CF was also identified to distinguish the stool microbiota of AD patients from healthy controls (decreased log_2_FC). No other bacterial biomarkers were found. Given the imbalance in sex distribution across study groups (controls: 60% female; AD and PD: 59.5% and 64.6% male, respectively), sex-stratified sensitivity analyses were performed. In the male-only subset (PD vs controls), two species (*Faecalicatena fissicatena* and *Phascolarctobacterium faecium*) were differentially abundant. While *F. fissicatena* was also detected in the full model, supporting the consistency of this association, *P. faecium* appeared specific to the male subset. In contrast, no significant taxa were identified in the female-only subset, likely reflecting reduced statistical power. These findings suggest that while individual species-level associations may vary by sex, the primary PD-associated microbiome patterns observed in the full model remain consistent. A direct comparison between AD and PD groups was performed; however, no species showed statistically significant differences between the two conditions. Beyond disease-related changes, *Bacteroidales bacterium* CF and *Holdemanella biformis* showed significant associations with sex (*p* < 0.05), whereas no taxa were associated with age. A boxplot summarizing the mean relative abundance ± standard error of differentially abundant species across patient groups is represented in **Figure 5**.

**Table 3:** Differential abundance of species across patient groups.

Comparison	Direction	Taxa	Log_2_FC	q-value
Control – PD	increases	*Ruminococcus* sp. JE7A12	+0.79	0.025
	decreases	*Faecalibacterium prausnitzii* *Agathobacter rectalis* *Roseburia intestinalis* *Faecalicatena fissicatena* *Holdemanella biformis* * Bacteroidales bacterium CF *	–0.68 –1.09 –0.81 –0.74 –0.59 –1.31	0.025 0.025 0.027 0.006 0.031 0.001
Control – AD	decreases	*Bacteroidales bacterium* CF *	–1.52	0.047

The table represents identified differentially abundant species (ANCOM-BC2) between controls vs. PD and controls vs AD. For each comparison, the direction of change (increase or decrease), log_2_-fold change (log_2_FC), and multiple-testing corrected significance (q-value) are reported. Asterisk (*) indicates species also significantly associated with sex (*p* < 0.05). No differentially abundant species were detected in the direct PD-AD comparison.

### 3.6 Disease duration and clinical scores association

In exploratory disease-stratified analysis (section 3.3), disease duration was identified as a key contributor to gut microbiota variation. In AD cohort, disease duration showed a modest but significant association with beta diversity (PERMANOVA, R^2^ = 0.045, *p* = 0.029), whereas cognitive function (MoCA score) showed a borderline association (R^2^ = 0.038, *p* = 0.077). Sex and age were not significantly associated with microbial composition in this cohort. In contrast, disease duration remained the strongest predictor of microbial variation in PD (PERMANOVA, R^2^ = 0.030, *p* = 0.013), with Hoehn & Yahr stage showing a significant association (R^2^ = 0.023, *p* = 0.035). MoCA score (R^2^ = 0.015, *p* = 0.438), constipation status (R^2^ = 0.010, *p* = 0.804), sex, and age were not significantly associated.

At species level, disease duration positively correlated with *Bacteroides uniformis* (Spearman ρ = 0.54, FDR = 0.096) and negatively correlated with *Peptococcus niger* (ρ = -0.55, FDR = 0.096) in AD patients. MoCA score was positively associated with *Faecalicatena fissicatena* (ρ = 0.52, FDR = 0.16) and *Paraprevotella clara* (ρ = 0.54, FDR = 0.16). In PD, no microbial species showed significance correlations with disease duration or cognitive function after FDR correction, indicating that progression-related changes are likely distributed across the microbiota rather than driven by single taxa. However, Hoehn and Yahr stage was linked with multiple taxa, including *Barnesiella intestinihominis* (ρ = -0.40, FDR = 0.18), *Blautia caecimuris* (ρ = 0.38, FDR = 0.18), *[Ruminococcus] torques* (ρ = 0.37, FDR = 0.18), and *Traorella massiliensis* (ρ = -0.38, FDR = 0.18). Within PD cohort, ANCOM-BC2 analysis adjusted for age and sex identified a limited number of taxa associated with constipation status. *Bacteroidales bacterium CF* was significantly depleted in constipated individuals (log_2_FC = −1.67, *q* = 0.003), whereas *Dialister invisus* showed increased abundance (log_2_FC = 0.92, *q* = 0.027).

## 4 Discussion

### 4.1 Study overview

In this study, we applied full-length 16S rRNA gene sequencing to characterize the gut microbiota at species-level resolution in Alzheimer’s disease, Parkinson’s disease, and age-matched healthy controls within a unified analytical framework. Our results demonstrate distinct microbiome alterations across neurodegenerative disorders, with more pronounced and consistent changes observed in PD compared with AD. Specifically, AD was associated with modest but statistically significant reduction in microbial diversity and richness compared with healthy controls, whereas PD showed no detectable changes in alpha diversity but exhibited significant compositional shifts. Beta diversity analysis confirmed that microbial community structure differed across groups, with PD contributing most strongly to this separation and sex emerging as an additional influencing factor. At species-level, PD was associated with a coherent pattern of differential abundance involving SCFA-producing taxa, while AD showed only minimal changes. Notably, a direct comparison between AD and PD groups revealed no significant species-level differences, potentially indicating that the observed microbial patterns should be interpreted as PD-associated rather than strictly disease-specific.

Taken together, these findings suggest that reduced gut microbial diversity may be more consistently associated with AD, whereas PD-associated microbiota alterations appear to be more heterogeneous and may reflect differences in disease phenotype rather than a uniform loss of microbial diversity (Vascellari et al., 2021; Zhang et al., 2023). Importantly, the observed significant differences in dispersion between group dispersion further suggests inter-individual variability, particularly within the PD cohort. This suggests that microbiome alterations in PD are not solely driven by consistent compositional shifts but may instead reflect multiple, patient-specific dysbiotic states. These findings support the concept of a personalized gut-brain axis, in which host genetics, diet, medication, and disease trajectory interact to shape microbiome configurations. This variability may partially explain inconsistencies across studies and highlights the importance of stratified and longitudinal approaches to better resolve microbiome patterns associated with neurodegenerative diseases.

### 4.2 Microbiome alteration in AD

Compared to PD, the AD cohort exhibited more modest and less consistent microbiome alterations. A small but significant reduction in microbial richness and diversity was observed, consistent with some prior studies, although findings across AD cohorts remain heterogeneous. A recent review have reported both decreased and unchanged diversity in AD-spectrum patients, highlighting variability likely driven by differences in study design, cohort composition, and analytical approaches (Li et al., 2024). At species level, only *Bacteroidales bacterium CF* was significantly reduced in AD compared with healthy controls. No additional taxa were identified, and no differences were detected in direct comparison between AD and PD. This limited signal may reflect the inherently heterogeneous nature of AD, which involves complex and multifactorial pathological processes, including amyloid-β deposition, tau pathology, neuroinflammation, and metabolic dysfunction (Dissanayaka et al., 2024).

Importantly, the relatively small sample size of the AD cohort represents a key limitation that may have reduced statistical power, increasing the likelihood of false-negative findings. Given the high within-group variability typical of AD populations, subtle microbiome changes may remain undetected in smaller cohorts. Consequently, the absence of broader species-level alterations should be interpreted cautiously and does not exclude a biologically relevant role of the microbiome in AD.

### 4.3 Microbiome alteration in PD

Our findings show a consistent depletion of SCFA-producing taxa in PD, including *Faecalibacterium prausnitzii*, *Agathobacter rectalis*, *Roseburia intestinalis*, and *Faecalicatena fissicatena*, alongside enrichment of *Ruminococcus* sp. JE7A12. These observations are consistent with previous studies describing reduced abundance of *Lachnospiraceae* and other butyrate-producing species in PD (Nishiwaki et al., 2022; Li et al., 2023; Zhang et al., 2023; Krueger et al., 2025). Because these taxa play an essential role in maintaining epithelial integrity and modulating intestinal inflammation, their depletion may reinforce proposed mechanisms linking gut dysbiosis, barrier dysfunction, and neuroinflammation in PD (Krueger et al., 2025). SCFAs, including butyrate, propionate, and acetate, are key metabolites produced through microbial fermentation of dietary fiber and play central roles in gut-brain axis signalling. Butyrate is essential for maintaining intestinal epithelial integrity, acting as the primary energy source for colonocytes and regulating tight junction expression (Fock and Parnova, 2023). In addition, SCFAs exert immunomodulatory effects through activation of G-protein-coupled receptors (GPR41, GPR43, and GPR109A) and inhibition of histone deacetylases (HDACs), leading to suppression of pro-inflammatory cytokine production and promotion of regulatory immune responses (Erny et al., 2015; Cao et al., 2025). Beyond local gut effect, SCAFs influence central nervous system function by modulating microglial maturation and activation states, thereby contributing to neuroimmune homeostasis. A reduction in SCFA-producing taxa may therefore contribute to increased intestinal permeability, systemic inflammation, and neuroinflammatory processes implicated in PD pathogenesis and α-synuclein aggregation (Harach et al., 2017; Chen and Lin, 2022). Propionate contributes to hepatic gluconeogenesis and metabolic signalling, while acetate plays a broader role in systemic energy metabolism and lipogenesis (Morrison and Preston, 2016). Although SCFA concentrations were not directly measured in this study, the depletion of SCAF-producing bacteria strongly suggests a functional shift.

Increase in *Ruminococcus* sp. JE7A12 levels further supports the concept that PD-associated microbiome alterations involve not only the loss of beneficial SCFA producers but also the expansion of taxa associated with mucin degradation and pro-inflammatory metabolic profiles. Although species-level evidence for *Ruminococcus* sp. JE7A12 remains limited, increased *Ruminococcus* abundances has been linked to disease duration and severity in previous studies (Mehanna et al., 2023), although findings remain inconsistent reflecting disease-related ecological restructuring rather than random fluctuation (Li et al., 2023). Among the differentially abundant taxa, *Faecalibacterium prausnitzii* is of particular interest due to its reported strong association with PD progression and neurodegenerative mechanisms (Vascellari et al., 2021; Li et al., 2023; Zhang et al., 2023). In a large metagenomic study, *F. prausnitzii* contributed disproportionately to the functional pathways differentiating PD from controls, and its depletion was linked to altered carbohydrate degradation, ribosomal genes enrichment, and markers of faster disease progression (Metcalfe‐Roach et al., 2024). The reduced levels observed in our PD group therefore align with prior evidence implicating this species in gut-brain axis alterations. Two taxa identified in our dataset (*Faecalicatena fissicatena* and *Bacteroidales bacterium CF*) are less frequently reported in the neurodegeneration literature. The depletion of *F. fissicatena* has not been consistently highlighted in PD cohorts, suggesting a potential novel player within the *Lachnospiraceae* family, while *Bacteroidales bacterium CF*, significantly decreased in both AD and PD patients, aligns with broader reductions in *Bacteroidetes* observed in neurodegenerative disorders (Guo et al., 2021; Liu et al., 2021; Li et al., 2023; Bai et al., 2024). Notably, the reported reduction withing PD patients with constipation in our study, further suggests a potential interaction between gastrointestinal dysfunction and microbiome composition. In contrast, *Holdemanella biformis* showed decreased abundances in our cohort, differing from previous studies (Zapała et al., 2021; Zeng et al., 2024). These discrepancies may reflect population-specific effects, disease heterogeneity, or methodological differences in taxonomic classification. Further targeted work will be needed to clarify the direction and functional implications of *H. biformis* in PD.

Overall, these findings support a model in which PD-associated gut dysbiosis is characterized by functional disruption rather than large-scale diversity loss, reinforcing the importance of species-level resolution.

### 4.4 Influence of clinical variables and demographics

Clinical and demographic variables contributed significantly to microbiome variability. Sex was associated with microbial community structure, consistent with previous evidence demonstrating sex-dependent differences in gut microbiome (Feng et al., 2014; Heravi et al., 2023). To further evaluate the impact of sex imbalance in our cohort, we performed sex-stratified analyses. In the male-only subset (PD vs controls), a limited number of taxa remained significant, including *Faecalicatena fissicatena*, which was also identified in the full model, supporting robustness of this association. In contrast, no significant taxa were detected in the female-only subset, likely due to reduced statistical power. These findings highlight the importance of including sex as a covariate in microbiome analysis.

Disease duration was significantly associated with microbial composition in both AD and PD, suggesting that microbiome alterations may evolve alongside clinical trajectory and may contribute to host immunometabolic changes influencing neuroinflammation and systemic homeostasis (Van Olst et al., 2021). In AD, common anaerobic gut commensal *Bacteroides uniformis* exhibited a positive correlation with disease duration, consistent with shifts in *Bacteroidetes* observed in aging and neurodegeneration (Koutsokostas et al., 2024). Conversely, *Peptococcus niger* showed a negative correlation with disease duration, although direct mechanistic links to cognitive decline remain to be established. In PD, disease duration explained a modest but significant proportion of beta diversity variance, while several taxa were associated with Hoen and Yahr stage (motor severity), including *Barnesiella intestinihominis* (negative), *Blautia caecimuris* (positive), *[Ruminococcus] torques* (positive), and *Traorella massiliensis* (negative). These taxa have been linked to disease progression and motor severity in previous studies. *Barnesiella* genus has been linked to faster PD progression and cognitive decline, being underrepresented in patients with rapid motor deterioration and showing negative correlations with cognition (Ren et al., 2020; Lubomski et al., 2022). *R. torques* correlates with PD-related clinical scores, suggesting its abundance may reflect overall disease severity rather than precise stage-specific changes (Li et al., 2019). While *T. massiliensis* and *B. caecimuris* have less direct evidence, their associations with clinical stage in our cohort indicate potential relevance for motor severity.

Cognitive performance (MoCA score) showed no significant association with microbiome composition in PD and only modest associations in AD. This supports its exclusion as a covariate, as it reflects disease severity rather than an independent confounder. However, *Paraprevotella clara* and *Faecalicatena fissicatena* were positively associated with MoCA score in AD; notably, *P. clara* has been reported in multi-omics AD studies as positively associated with AD severity, suggesting it may track cognitive performance alongside disease progression (Meng et al., 2024). In PD, no individual species reached significance for correlations with MoCA after FDR correction, implying that progression-related shifts are likely distributed across the microbial community rather than concentrated in single taxa.

Together, these findings underscore that gut microbiome alterations may not only distinguish disease states but also reflect clinical trajectory, reinforcing the potential of microbial signatures as progression biomarkers. Nonetheless, causal relationships and functional implications remain to be established in larger, longitudinal cohorts incorporating integrated multi-omics and mechanistic interrogation.

### 4.5 Impact of pharmacological treatment

Pharmacological treatment represents a major potential confounder, particularly in PD, where medications such as levodopa, dopamine agonists, and monoamine oxidase inhibitors can directly influence gut microbiota composition and metabolism (Van Kessel et al., 2019; Palacios et al., 2021). Microbial metabolism of levodopa may affect both drug bioavailability and microbiome composition, introducing complex bidirectional interactions between host, microbiome, and treatment (Van Kessel et al., 2019). Although detailed pharmacological metadata were collected, incorporation of non-collinear medication classes into multivariate models did not alter the association between disease status and microbiome composition. Medication classes strongly associated with disease status were excluded from the multivariate model due to collinearity, whereas more evenly distributed treatments (e.g. statins and NSAIDs) were retained. Adjustments for these variables did not alter the significant association between disease status and microbiome composition.

These findings suggest that observed microbiome differences are not solely driven by pharmacological treatment, although residual confounding cannot be excluded.

### 4.6 Limitations and future directions

We acknowledge the presence of several limitations in the present study. The cross-sectional design limits our ability to infer causal relationships between gut microbiota alterations and neurodegenerative disease, and the observed associations cannot determine whether microbiome alterations contribute to disease pathogenesis or arise because of disease progression, medication use, or lifestyle factors. Longitudinal studies tracking individuals from prodromal stages, mechanistic *in vitro* or animal models, and interventional studies targeting microbial composition will be critical to clarify these temporal and functional relationships. The relatively small and unbalanced AD cohort may have reduced statistical power, increasing the likelihood of false-negative findings. High inter-individual variability may further obscure subtle microbial differences. Additional limitations include the absence of dietary habits, incomplete pharmacological metadata, and lack of direct metabolite measurements. Dietary patterns and nutrient intake are major determinants of gut microbiome composition and have been significantly associated with gut microbiota shifts and predicted functional pathways in PD, highlighting the potential influence of diet on microbial variation (Kwon et al., 2024). This is particularly relevant in neurodegenerative diseases, where dysphagia in PD or cognitive decline in AD may alter dietary patterns (Cipriani et al., 2016; Ueha et al., 2023). Future longitudinal studies should incorporate dietary assessments to better distinguish disease-related from diet-driven microbiota changes. Concerning gastrointestinal motility, constipation status was assessed in PD patients and included in our analyses. Nonetheless, stool metrics (e.g., Bristol stool scale scores, frequency of bowel movements) were not collected, which limits the granularity of our assessment and remains a relevant consideration when interpreting microbiota changes in relation to PD pathophysiology. Finally, the predominantly homogeneous ethnic homogeneity (white ethnicity) may limit generalizability of the results to more diverse populations.

Despite these limitations, the study possesses several strengths. The use of high-resolution 16S rRNA amplicon sequencing enabled species-level resolution, allowing the characterization of subtle but biologically meaningful microbial shifts. The inclusion of both AD and PD patients alongside age-matched healthy controls enabled direct comparison within the same cohort, thereby reducing potential confounding due to differences between independent study populations. Moreover, the analytical framework accounted for key demographic covariates, further supporting the robustness of the observed disease-associated microbiome alterations. Future studies should integrate longitudinal sampling, detailed clinical and pharmacological metadata, and multi-omics approaches, including metabolomics and metatranscriptomics, to better link microbial composition with functional outputs. Such approaches will be critical for determining whether microbiome alterations represent viable biomarkers or therapeutic targets in neurodegenerative diseases.

## 5 Conclusion

Using full-length 16S rRNA gene sequencing we characterized the gut microbiota in AD, PD, and age-matched healthy controls within a unified analytical framework. Our results reveal distinct microbiome alterations associated with neurodegenerative disease, with more consistent and pronounced changes observed in PD compared with AD. PD was characterized by a depletion of SCFA-producing taxa, including members of the *Faecalibacterium*, *Agathobacter*, *Roseburia*, and *Faecalicatena* genera, alongside enrichment of *Ruminococcus* sp. JE7A12. These alterations are consistent with proposed mechanisms linking gut dysbiosis, impaired epithelial barrier function, and neuroinflammation in PD. In contrast, AD exhibited only modest reductions in microbial diversity and limited species-level shifts, supporting the view that gut microbiota alterations in AD are more subtle and heterogeneous. No species-level differences were detected between AD and PD in direct comparison, indicating that the identified taxa represent PD-associated patterns rather than strictly disease-specific signatures. Associations between sex and clinical variable further highlight the importance of accounting for biological and clinical heterogeneity in microbiome studies.

Taken together, these findings reinforce PD as the neurodegenerative condition most robustly associated with gut microbiota dysbiosis and demonstrate the added value of species-level profiling for identifying biologically and clinically meaningful microbial signatures. This is consistent with previous work demonstrating that microbiome-based diagnostics performance is highest at species level and declines at broader taxonomic ranks (De Jaegher et al., 2025), underscoring the importance of high-resolution approaches. Overall, high-resolution microbiome profiling may therefore contribute to future mechanistic studies and support the development of microbiome-informed biomarkers or therapeutic strategies in neurodegenerative disorders.

## Data Availability

The datasets presented in this study can be found in online repositories. The names of the repository/repositories and accession number(s) can be found below: https://www.ebi.ac.uk/ena/browser/view/PRJEB106350.
